# Structure and Strength of Iron-Copper-Carbon Nanotube Nanocomposites

**DOI:** 10.1186/s11671-016-1298-8

**Published:** 2016-02-09

**Authors:** Oleh Boshko, Mykola Dashevskyi, Olga Mykhaliuk, Kateryna Ivanenko, Smail Hamamda, Sergiy Revo

**Affiliations:** Taras Shevchenko National University of Kyiv, 64/13, Volodymyrska Street, 01601 Kyiv, Ukraine; Laboratory of Thermodynamics and Surface Treatment of Materials, University of Frères Mentouri Constantine 1, B.P. 325 Route Ain El Bey, Constantine, 25017 Algeria

**Keywords:** Composite materials, Powder metallurgy, Crystal structure, X-ray diffraction, Thermopower, Tensile strength

## Abstract

Nanocomposite materials of the Fe-Cu system with/without small addition of carbon nanotubes have been synthesized by mechanochemical activation of elemental Fe and Cu powders in a high-energy planetary ball mill and have been examined by the X-ray diffraction method, SEM and the thermopower methods; the tensile strength of the materials obtained has been estimated. The metastable (Fe, Cu) supersaturated solid solution is formed in the Fe-Cu nanocomposites during milling process. The coherent scattering block size of the materials obtained is decreased with increase of milling time. The duration of mechanochemical activation affects the physical properties of nanocomposites studied. Addition of a small amount of nanotubes into Fe-Cu charge results in a significant increase of strength of the Fe-Cu (4:1) + CNT nanocomposite materials (NCMs) obtained.

## Background

Our analysis of information on production methods for high-strength metallic materials, described in the current scientific literature, brings us to the conclusion that, as a rule, such materials are obtained by reducing the size of their grains. In this respect, research works on new nanomaterials and development of methods for their production are topical. Formation of a predetermined micro- and nanocrystalline material structure and creation of various composites based thereon opens up vast possibilities of increasing strength properties of products made of these materials and allows extending their functionality. Refinement of grain size of metallic blanks, comprising two or several components, allows producing a nanocomposite material (NCM) with high properties of practical value [1]. Such components can be represented by stacked alternating foils of dissimilar and, in most cases, insoluble metals, whose pseudoalloys or powder mixtures are used to form NCM after pressing, sintering and rolling. For these NCM, the ultimate tensile stress (*σ*_B_) increase with reduction of grain size (in this case, reduction of thickness of layers or lamels (*h*)) is governed by the Hall-Petch equation [2] with additional contribution of surface tension force:

$$ {\sigma}_{\mathrm{B}}={\sigma}_0+{k}_{\mathrm{b}}{h}^{-\frac{1}{2}}+\frac{\gamma_{\mathrm{C}}}{h}{A}_0, $$

where *σ*_0_ is the flow stress, *k*_b_ is a blocking factor of dislocations by structural barriers, *γ*_C_ is the coefficient of linear tension on boundaries of adjacent lamels and *A*_0_ is a parameter, which takes into account lamel orientation about the axis of elongation.

In addition to the grain size, a substantial factor affecting the physicomechanical properties of the materials discussed here is introduction of high density of dislocations in cold rolling of composite systems. A distinct feature that arises for NCM is a unique combination of high strength and plasticity. A typical example of a dramatic increase in strength is Fe-Cu nanocomposite material, where sheets of this composite are almost 1.85 times greater than the value of *σ*_B_ of its counterpart pseudoalloy [1]. The production method for such composites comprises mixing its component powders, pressing and sintering a composite blank (operations of precursor production) and its further pressing for obtainment of a nanoscaled lamellar structure and target physicomechanical properties.

The objective of this work is to extend the possibilities of the above-mentioned NCM production method owing to the use of a planetary-type ball mill for production of precursors. Another objective of this study is analysis of nanocomposite structure formation mechanisms and influence of the structure features on the physicomechanical properties of the produced materials. We have chosen to employ such a mill for obtainment of precursors because powder grains are not only ground in the mixing process of the composite components in the mill but also their physicochemical characteristics become altered as well. When powders are treated in the mill, particles of the material being ground collide multiple times with other particles, with the grinding balls and the walls of the mill’s bowls. The particles become mechanically activated, their reactivity is raised and even poorly soluble components become mutually mechanically alloyed owing to these collisions at high acceleration values of the bowls. Effectiveness of treatment of powders in such mills depends on a number of performance characteristics of these mills. These include, in the first place, power of a mill, design features, etc. Some authors [3, 4] believe that effectiveness of a mill operation is mainly determined by absolute particle production rates, while others assign primary importance to other technical characteristics: volume of the bowl, diameter of the grinding balls, material of the balls, spinning rates of the drum plate and the mill’s bowl, ratio of those rates, etc. It is evident that all these characteristics are directly linked to the accelerations (*g*) affecting the material in the mill’s bowls and this is the parameter that needs to be taken into account in solving the assigned tasks. The value of *g* does not exceed 20*g* for commonly used mills. In this situation, the material needs to undergo many hours of treatment to achieve an effect on the material being ground [5–8]. In our study, the planetary mill used to produce the precursors is capable of acceleration up to 50*g*. This has enabled us to reduce the time needed for treatment of the powders and to improve its effectiveness for achievement of high physicomechanical properties of NCM.

## Methods

Specimens of the NCM were produced from the following components: PMS1 Cu powder [9], PZ1 iron [10] and multiwall carbon nanotubes (MCNT) obtained by CVD method in a rotating reactor [11, 12]. The mean diameter of the carbon nanotubes was 10–20 nm; their specific surface area, which was determined by argon desorption, was 200–400 m^2^/g; and their bulk density varied from 20 to 40 g/dm^3^. MCNT were added to the Fe-Cu mixture at the amount of 0.5–2 vol.%. The properties of Fe-Cu-MCNT nanocomposite material were compared with the properties of Fe specimens and Cu specimens, as well as with the properties of Fe composites with Cu, Fe-MCNT and Cu-MCNT, produced by the same method as was used for production of Fe-Cu-MCNT nanocomposite materials. The proportions of Fe and Cu in the respective mixtures were varied as 2:1, 4:1 and 6:1. The source substance powders were mixed in the same proportions and treated in cycles (the cycle time was 5 min) in the planetary-type ball mill (acceleration—50*g*, pressure on a substance particle—5 GPa). Twenty grinding balls made of a hard alloy were used in each of the three bowls of the mill. The obtained mixtures were pressed at the pressure of 30 GPa. Then, the compressed specimens were annealed in argon medium during 30 min at 950 °C and rolled into 1.5–2-mm-thick sheets at room temperature. The total reduction of the precursors was 80–85 %. Rolling was alternated with annealing in the above-mentioned conditions. Sheets of the produced material were used to make specimens for the study. The tensile strength was calculated on specimens with the working area of approximately 20 mm long and 4–5 mm wide at room temperature in the air and at the tensile rate of 5 mm/min. Ten specimens were used to find the average *σ*_В_.

SEM exanimation of the samples was carried out using a scanning electron microscope JEOL JSM-840, equipped with a system of X-ray microspectroscopy analysis (LINK add-on unit to electron microscope).

Since the differential thermopower resultant from deformation (*E*_T_) gives an adequate representation of changes in the density of the substances’ crystalline structure defects, arising from deformation and annealing [13], this method has been chosen in this study for analysis of structural changes in the NCM specimens after the mentioned actions. DRON-4.0-automated X-ray unit was used to obtain X-ray diffraction patterns of the substances’ specimens, using filtered cobalt X-rays *K*α = 1.7909 Å (at a discrete mode). Positions of the peak centres of gravity were calculated with the relative error of ±0.001°–0.005° and integral intensities with the error of ±5–15 % [14].

## Results and Discussion

Enhanced characteristics of the NCM specimens, obtained by pressing the powder mixtures and rolling the nanocomposite materials, were achieved owing to uniform distribution of its components in the material volume, dispersion of MCNT agglomerates and mutual mechanical alloying of iron and copper in the planetary ball mill. At the same time, cyclic treatments of the component mixture in the planetary mill provide for a fine-grained structure in the NCM produced, while enhanced physicomechanical properties of the carbon nanotubes, introduced into the composition, provide for strengthening of the nanocomposite material in a number of cases. This hardening of composites after the precursor rolling occurs due to blocking of dislocation motions by the grains’ boundaries, as well owing to a uniform distribution of the components over the material’s volume. Apart from downsizing and mechanical activation of Fe and Cu powder particles used for making the precursors, the ball-milling process leads to mutual dissolution of iron and copper with formation of solid solutions of iron in copper and copper in iron.

**Fig. 1:**
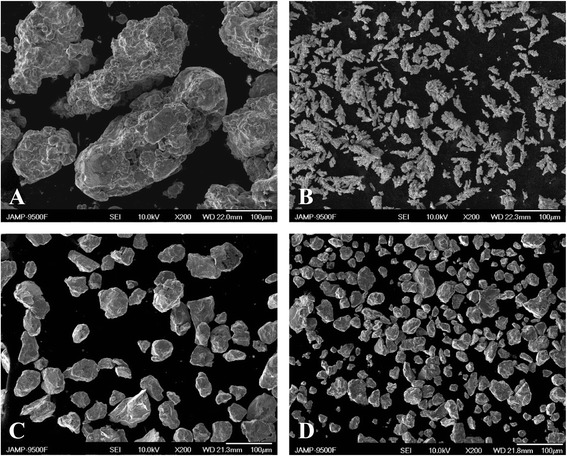
SEM of Cu (**a**) and Fe (**b**) input powders, powder mixture Fe-Cu (4:1) treated in the planetary mill during 120 min (**c**) and Fe-Cu (4:1)-MCNT powder mixture (1 vol.%) treated in the planetary mill during 120 min (**d**)

Figure 1 presents photographs of appearance of the original iron and copper powders (Fig. 1a, b), mixture Fe-Cu (Fig. 1c) and as part of a mixture with MCNT after treatment in the planetary mill during 120 min (Fig. 1d). These pictures demonstrate that the treatment alters both the morphology and the dispersion (dimensions of the powder particles’ peak cross section for this fraction (*d*)). After the initial treatment (up to 20 min), the dimensions of *d* are reduced virtually in all the particles and with the increase of the treatment time from 20 to 120 min, individual particles get agglomerated into small complexes and possibly sintered. Adding MCNT into the powders acts to a certain extent as a blocking factor to formation of agglomerates and improves uniformity of the powder particle distribution over the dimensions (see Fig. 1d).

It needs to be noted that the size of the coherent scattering blocks, which was calculated from the X-ray diffraction data, is much smaller than the size of the particles shown on Fig. 1. As such, their size for the treated copper is only 20–30 nm.

**Fig. 2:**
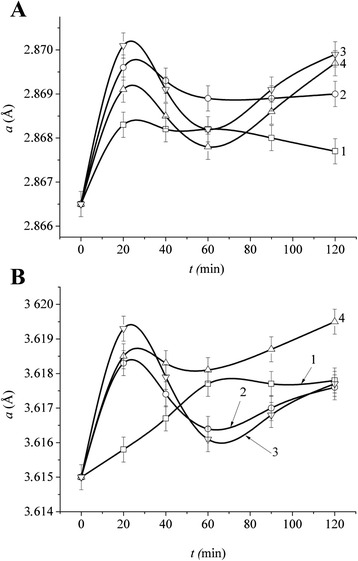
Variation of the lattice parameters of iron (**a**) and copper (**b**) in the rolled specimens after mechanochemical treatment of Fe and Cu powder mixtures (*1*–*3*) and Fe-Cu-MCNT (1 vol.%) (*4*) for the following proportions of Fe and Cu components: 2:1 (*1*); 4:1 (*2*, *4*); and 6:1 (*3*) against their treatment time

In addition to this, the X-ray study has shown that interaction of Fe and Cu powders in the NCM component mixing process leads to formation of metastable solid solutions of iron in copper and copper in iron. The parameters of both iron and copper lattices (see Fig. 2a, b) are varied, as a rule, non-monotonously with the increase of the treatment time of the powder mixture (*τ*).

The most considerable increase of the parameters in the majority of the specimens was observed at the *τ* ≤ 20 min. This is characteristic of intense dissolution of iron in copper and copper in iron, accompanied by formation of corresponding metastable solid solutions. The parameters of the lattices are usually decreased as a result of the increase of the mixture treatment time in the range of *τ* = 20–60 min and are increased again with *τ* = 60–120 min, in most cases not attaining the value corresponding to *τ* = 20 min.

**Fig. 3:**
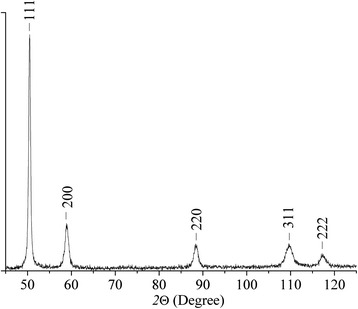
X-ray diffraction pattern of the precursors made of a mixture of Fe and Cu powders, proportioned as 3:7, after treatment in the planetary-type mill during 120 min

It is significant that no X-ray reflections from the iron bcc lattice were noticed when the iron and copper powders were mixed during *τ* < 120 min, Fe/Cu powder proportions being 3:7, 2:8 and 1:9. As such, Fig. 3, for example, shows an X-ray pattern fragment for Fe and Cu precursors proportioned as 3:7. This proves formation of an oversaturated solid solution. Additionally, the material made by pressing these mixtures is not ferromagnetic, which is a piece of evidence of Fe-phase absence in the precursors.

The presence of the nanotubes, although being an obstacle for formation of the powder particle agglomerates during the initial treatment stages, in the long run does not impede formation of metastable solid solutions of Fe and Cu. This is proven by the alteration of their lattices’ parameters (curve 4 on Fig. 2a, b) in the Fe-Cu system with 4:1 concentration of the components.

Thus, the target structure of the rolled precursors can be implemented depending on the degree of mechanochemical activation of the powder mixture and, consequently, the properties of the precursors can be optimized. Notwithstanding limited solubility of the metals’ components in the equilibrium state [15], almost the total amount of iron (within the framework of this study), used for preparation of the precursors, can be dissolved in copper, whose crystalline lattice parameter is greater than the respective parameter of iron (*a*_Cu_ = 3.6150 Å; *a*_Fe_ = 2.8665 Å), during mechanical alloying and provided the treatment time in the planetary mill is sufficient.

**Fig. 4:**
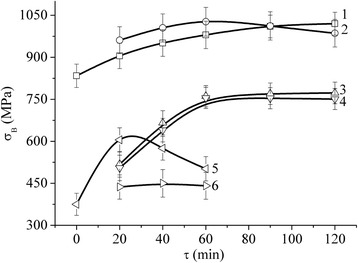
Tensile strength *σ*_В_ of the rolled precursor Fe (*1*); Fe-MCNT (0.5 vol.%) (*2*); Fe-MCNT (1 vol.%) (*3*); Fe-MCNT (1.5 vol.%) (*4*); Cu (5); and Cu-MCNT (0.5 vol.%) (*6*) as a function of the treatment time of the respective component mixtures in the planetary mill

The study results of NCM strength properties have enabled us to establish that their value of *σ*_В_ depends on both the total treatment time in the planetary mill and on the component concentration in the mixtures. Thus, for M1 sheet copper (100-μm-thick copper foil), *σ*_В_ equals 314 ± 22 MPa and for a copper sheet pressed from PMS1 powder and rolled at room temperature with 80 % reduction, *σ*_В_ = 382 ± 24 MPa, whereas for the precursor rolled from the copper powder (the optimal treatment time in the planetary mill *τ* = 20 min), the value of *σ*_В_ = 605 ± 31 MPa. The strength of an iron sheet pressed from PZ-1 powder and rolled at room temperature with 80 % reduction is *σ*_В_ = 834 ± 42 MPa, while the same value for the rolled precursors is *σ*_В_ = 1020 ± 41 MPa. The presence of the nanotubes in iron or copper powders does not produce any significant effect on the strength of the rolled precursors (see Table 1, Fig. 4).

**Table 1:** Peak values of the tensile strength (*σ*_В_) of the produced specimens, which contain MCNT, treated during different time periods in the planetary mill

Material	Content of MCNT (vol.%)									
	0		0.5		1		1.5		2	
	Treatment time in the planetary mill (min)									
	20	120	20	60	20	60	60	120	60	120
	*σ*_В_ (MPa)									
Fe-Cu (2:1)-MCNT	755 ± 41	–	–	700 ± 39	672 ± 44	–	968 ± 45	–	850 ± 42	–
Fe-Cu (4:1)-MCNT	–	867 ± 43	1452 ± 53	–	–	1800 ± 40	–	1130 ± 47	–	920 ± 46
Fe-Cu (6:1)-MCNT	858 ± 43	–	–	1108 ± 45	–	849 ± 42	755 ± 47	–	–	–
Fe-MCNT	–	1020 ± 41	–	1027 ± 51	–	761 ± 38	–	755 ± 38	–	–
Cu-MCNT	605 ± 43	–	–	441	–	–	–	–	–	–

**Fig. 5:**
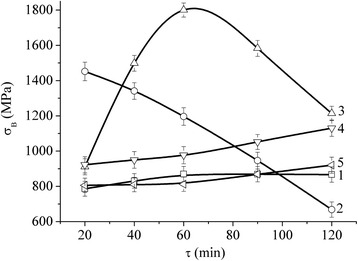
Tensile strength *σ*_В_ of the rolled Fe-Cu precursor 4:1 (*1*); Fe-Cu (4:1)-MCNT (0.5 vol.%) (*2*); Fe-Cu (4:1)-MCNT (1.0 vol.%) (*3*); Fe-Cu (4:1)-MCNT (1.5 vol.%) (*4*); and Fe-Cu (4:1)-MCNT (2.0 vol.%) (*5*) as a function of the treatment time of the respective component mixtures in the planetary mill

**Fig. 6:**
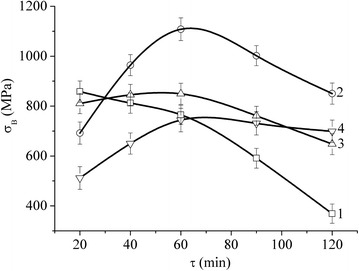
Tensile strength *σ*_В_ of the rolled Fe-Cu precursors 6:1 (*1*); Fe-Cu (6:1)-MCNT (0.5 vol.%) (*2*); Fe-Cu (6:1)-MCNT (1.0 vol.%) (*3*); and Fe-Cu (6:1)-MCNT (1.5 vol.%) (*4*) as a function of the treatment time of the respective component mixtures in the planetary mill

At the same time, the presence of the nanotube produces more significant effect for the composites. As is seen in Table 1, the concentration of iron in Fe-Cu-MCNT nanocomposite material affects its *σ*_В_ value to a lower extent than the content of nanotubes and the treatment time of the components in the mill. Thus, *σ*_B_ is increased from *σ*_B_ = 755 ± 41 MPa to 858 ± 43 MPa with the increase of iron to copper proportion from 2:1 to 6:1 and *τ* = 20 min (i.e. almost a 20 % increase). At the same time, the increase of the powder treatment time for one and the same concentration of the components leads to an increase (or a decrease) of *σ*_B_ to different extents, depending on the percentage of MCNT (see Figs. 5 and 6). The peak value of *σ*_B_ has been obtained for NCM with Fe/Cu proportion of 4:1 and with MCNT content of ~1 vol.% during the treatment time of *τ* = 60 min. This value is *σ*_В_ = 1800 ± 40 MPa.

The analysis of Figs. 4, 5 and 6 leads us to the conclusion that the maximum of the treatment time effectiveness against the value of *σ*_В_ of the rolled precursors is observed for the specimens with Fe and Cu component concentration of 4:1, with MCNT content of 1 vol.% (see Figs. 5 and 6).

In order to establish the influence of the behaviour of the specimens’ structural properties in deformation and annealing on the value of *σ*_В_, we have analysed how their thermopower (*E*_T_) varies in rolling and thermal treatment.

Thermopower of metals depends on a number of factors. This is, first of all, temperature, Fermi surface properties, electron scattering behaviour on impurities and defects of the crystalline lattice [16]. When the differential thermopower is studied on a standard-specimen thermocouple, where the specimen can be, in particular, a material, whose structural state is different from the standard only by its type or degree of treatment (specimen deformation or mode of specimen thermal treatment), the value of *E*_T_ will reflect the changes caused by the treatment. These can be changes of the phase or impurity content in the specimens, defects of their structure and others. Defects of various types in metals and more importantly for the results of our study—in NCM—are caused by plastic deformation. All of them, however, block motion of the electric charge, change the phonon spectrum and eventually affect kinetic, particularly thermoelectric, characteristics of the materials.

**Fig. 7:**
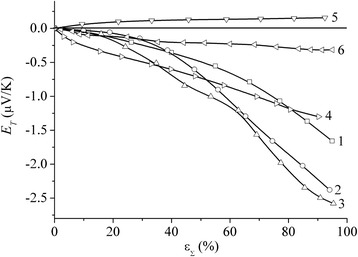
Thermopower of Fe-Cu 4:1 and Fe-Cu 2:1 specimens treated in the planetary mill during 20 (*1*) and 120 (*2*, *3*) minutes; Fe-Cu 2:1 (*4*) multilayer NCM pressed from PMS1 copper powder (*5*) and Armco Fe (*6*) plotted against the degree of the total relative rolling deformation

The analysis of the study results of multilayer NCM, including the ones produced from iron and copper powder mixtures using the thermopower method, shows that the characteristics of their properties in relation to the respective characteristics of their components are nonadditive [1]. No additivity of characteristics was observed for the similar composites produced from Fe and Cu powders with treatment in the planetary mill. Additivity of the characteristics was observed neither in their strength (see Table 1, Figs. 4, 5 and 6) nor in the dependencies of deformation-induced thermopower (Fig. 7).

At the same time, if for pure metals dependencies of *E*_T_ = *f*(*ε*_Σ_) attain saturation (see curves 5 and 6, Fig. 7), for both multilayer NCM and composites obtained by mechanochemical treatment, no such behaviour of dependencies was observed. Since variation of *E*_T_ with the increase of the deformation density is an adequate reflection of variation in the density of defects of the specimens’ crystalline structure, the nonmonotonous variation of *E*_T_ = *f*(*ε*_Σ_) dependencies must be an indication of nonmonotonous variation of the defect density in the specimens. Their deformation areas, where the value of *E*_T_ is virtually unchanged, may reflect dislocation ordering, formation and strengthening of a distinctive dislocation texture. Neither for Fe-Cu multilayer NCM nor for the specimens of composites obtained using mechanochemical treatment *E*_T_ = *f*(*ε*_Σ_) dependencies reach saturation (see curves 1–3, Fig. 7). At the same time, *E*_T_ reaches greater values (see curves 1–3, Fig. 7) than for layered NCM, which may be an indication of more effective blocking of dislocation mobility by the grains’ boundaries in these specimens. This, in its turn, is the reason for their higher physicomechanical characteristics mentioned above.

**Fig. 8:**
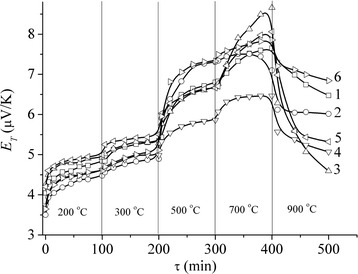
Thermopower of Fe-Cu (4:1) precursor specimens rolled with relative deformation of *ε*_Σ_ = 70 (*1*, *3*, *5*) and 95 (*2*, *4*, *6*) % treated in the planetary mill during 20 (*1*, *2*), 60 (*3*, *4*) and 120 (*5*, *6*) minutes against the time of step-by-step annealing at the temperature values given on the figure

The structural state of the material changes as a result of annealing. The analysis of *E*_T_ = *f*(*ε*_Σ_)_*T* = const_ curves shown on Fig. 8, where *τ* is the total annealing time, provides an evaluation of the time needed for relaxation of voltages, structural defect annihilation as mainly vacancies at *Т* ≤ 300 °С and dislocations at *Т* = 500–700 °С, for redistribution of impurities and consequently structure transition from one pseudoequilibrium state into another at *Т* > 700 °С.

**Fig. 9:**
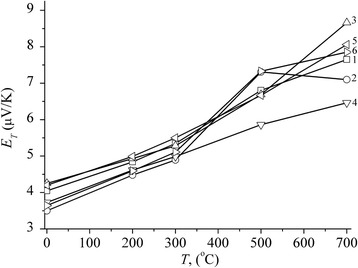
Thermopower of Fe-Cu (4:1) precursor specimens rolled with relative deformation of *ε*_Σ_ = 70 (*1*, *3*, *5*) and 95 (*2*, *4*, *6*) % treated in the planetary ball mill during 20 (*1*, *2*), 60 (*3*, *4*) and 120 (*5*, *6*) minutes as a function of the temperature of isochronous multiple annealing (*τ* = 100 min)

*E*_T_ = *f*(*Т*) curve as it is seen from Fig. 9 demonstrates almost linear dependence. This certifies that structural relaxation for such systems is similar to the one for amorphous alloys.

## Conclusions

We have developed a method for production of a new nanocomposite material comprising the following components: iron, copper and multiwall carbon nanotubes with a high ultimate tensile strength. The most promising result of our study is that *σ*_B_ is nonadditive to the strength of its components (sheet iron and copper) and is approximately three times greater than the latter.

We have established that high-energy treatment of NCM powder components in the planetary mill leads to consistent changes in the size of the cross section of the particles being treated and mutual dissolution of iron and copper (formation of metastable supersaturated solid solutions), whereas no limitations have been found for solubility of iron in copper. The sizes of coherent scattering blocks are usually decreased after the mechanochemical activation of the respective mixtures in the mill, while the lattice parameters of both iron and copper have changed in accordance with the time of the mixtures’ treatment in the mill.

The presence of the nanotubes prevents the formation of agglomerates on the first stages of milling and at the same time prevents the mutual solution of powders. This is proven by the alteration of their lattice parameters. At the same time, cyclic treatments of the component mixture in the planetary mill provide for a fine-grained structure of the NCM produced, while enhanced physicomechanical properties of the carbon nanotubes, introduced into the composition, provide for strengthening of the nanocomposite material in a number of cases.

We have established the optimal modes for the mechanochemical activation and proportions for the NCM powder mixture component concentrations to achieve the target structure of the rolled precursors, as well as for obtainment of composites with maximal ultimate tensile strength value. The maximum of *σ*_В_ = 1800 ± 40 MPa has been obtained for Fe-Cu-MCNT (1 vol.%) composite with Fe:Cu proportion of 4:1 after activation of the components in the mill during 60 min and the precursor rolling with relative reduction of 80–85 %.

Based on the analysis of the thermopower (*E*_T_) variation during the studied precursors’ rolling, we have shown that the increase of *σ*_B_ is caused by an increase of the dislocations’ density in the specimens, which is greater than the density of similar rolling of Fe-Cu multilayer composite material. This is a piece of evidence of more effective blocking of dislocations’ mobility by the grain boundaries in the specimens produced by the mechanochemical activation and provides a higher *σ*_B_ for them.
